# DAMe: a toolkit for the initial processing of datasets with PCR replicates of double-tagged amplicons for DNA metabarcoding analyses

**DOI:** 10.1186/s13104-016-2064-9

**Published:** 2016-05-03

**Authors:** Marie Lisandra Zepeda-Mendoza, Kristine Bohmann, Aldo Carmona Baez, M. Thomas P. Gilbert

**Affiliations:** Evogenomics, Centre for GeoGenetics, Natural History Museum of Denmark, University of Copenhagen, Øster Voldgade 5-7, 1350 Copenhagen, Denmark; Undergraduate Program on Genomic Sciences, Center for Genomic Sciences, National Autonomous University of Mexico (UNAM), Av. Universidad s/n Col. Chamilpa, 62210 Cuernavaca, Morelos Mexico

**Keywords:** Demultiplexing, DNA metabarcoding, Double-tagged amplicons, Environmental DNA, High throughput sequencing, Tag jumping

## Abstract

**Background:**

DNA metabarcoding is an approach for identifying multiple taxa in an environmental sample using specific genetic loci and taxa-specific primers. When combined with high-throughput sequencing it enables the taxonomic characterization of large numbers of samples in a relatively time- and cost-efficient manner. One recent laboratory development is the addition of 5′-nucleotide tags to both primers producing double-tagged amplicons and the use of multiple PCR replicates to filter erroneous sequences. However, there is currently no available toolkit for the straightforward analysis of datasets produced in this way.

**Results:**

We present DAMe, a toolkit for the processing of datasets generated by double-tagged amplicons from multiple PCR replicates derived from an unlimited number of samples. Specifically, DAMe can be used to (i) sort amplicons by tag combination, (ii) evaluate PCR replicates dissimilarity, and (iii) filter sequences derived from sequencing/PCR errors, chimeras, and contamination. This is attained by calculating the following parameters: (i) sequence content similarity between the PCR replicates from each sample, (ii) reproducibility of each unique sequence across the PCR replicates, and (iii) copy number of the unique sequences in each PCR replicate. We showcase the insights that can be obtained using DAMe prior to taxonomic assignment, by applying it to two real datasets that vary in their complexity regarding number of samples, sequencing libraries, PCR replicates, and used tag combinations. Finally, we use a third mock dataset to demonstrate the impact and importance of filtering the sequences with DAMe.

**Conclusions:**

DAMe allows the user-friendly manipulation of amplicons derived from multiple samples with PCR replicates built in a single or multiple sequencing libraries. It allows the user to: (i) collapse amplicons into unique sequences and sort them by tag combination while retaining the sample identifier and copy number information, (ii) identify sequences carrying unused tag combinations, (iii) evaluate the comparability of PCR replicates of the same sample, and (iv) filter tagged amplicons from a number of PCR replicates using parameters of minimum length, copy number, and reproducibility across the PCR replicates. This enables an efficient analysis of complex datasets, and ultimately increases the ease of handling datasets from large-scale studies.

**Electronic supplementary material:**

The online version of this article (doi:10.1186/s13104-016-2064-9) contains supplementary material, which is available to authorized users.

## Findings

### Background

DNA metabarcoding is a powerful tool for the simultaneous identification of multiple taxa within an environmental sample through PCR amplification and sequencing of amplicons generated with primers that are specific for the taxonomical group of interest [1]. Prior to the advent of high-throughput sequencing (HTS), most DNA-based studies used PCR-based amplicons that were directly Sanger-sequenced [2] or hybridized [3], thus the scope was limited to the generation of relatively few sequences per sample. More recently, HTS platforms have been adapted into powerful and economic means for generating large datasets from many samples in parallel (e.g. [4–7]).

DNA extracts from the samples are typically PCR-amplified using primers with 5′-nucleotide tags, enabling the simultaneous sequencing of a large number of samples. It is recommended to tag amplicons at both extremities and to make more than one PCR replicate [8]. Although coupling metabarcoding with sequencing in a HTS platform considerably reduces the time-consuming steps of data generation per sample [1], it also confers challenges such as (i) handling of large datasets [9], (ii) identifications of erroneous sequences [10], and (iii) generation of tags with un-used tag combinations [11]. Given that the size of the generated datasets can be very large, they can only be handled through computational toolkits that perform the necessary basic processing of the raw data [9].

This initial processing is a key step with regards to data quality and can have serious implications on subsequent taxonomic assignment [12]. It is important to consider that different kinds of errors can originate both in the laboratory and during sequencing, thus various aspects have to be carefully addressed in this pre-taxonomy assignment phase [13]. For instance, potential cross-contamination between samples, tagged primers and tagged amplicons can occur during the initial PCR tagging step, and errors in the amplified sequences can arise due to base misincorporations and chimeric sequence formation [14, 15]. Once sequenced, these errors may overinflate biodiversity estimates [12]. Furthermore, HTS platforms produce sequences with specific patterns of nucleotide miscalling and insertion/deletion rates, which may result in different community profiles [16]. Another major challenge is the handling of datasets from protocols that require the pooling of multiple tagged amplicons prior to the so-called library building (preparation for sequencing) [17]. These sequencing libraries are often subject to blunt-ending and a final round of index-PCR amplification that can result in the so-called tag jumping [8], meaning that sequences with unused tag combinations derived from used tags are formed [18, 19]. This problem is rarely acknowledged in the metabarcoding literature, yet may lead to incorrect assignment of sequences to samples and artificially inflate diversity, unless the sequences can be identified and excluded from downstream analyses [8].

**Fig. 1:**
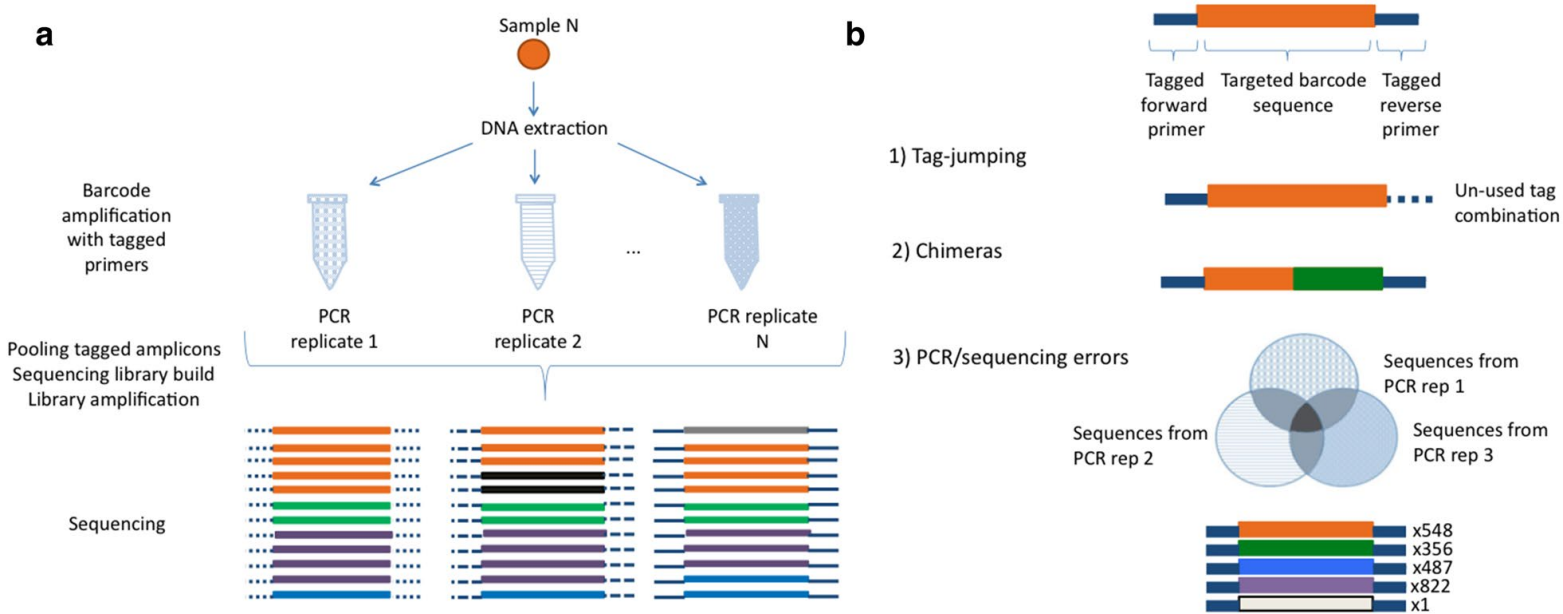
Laboratory setup. In the scheme each unique sequence has a different colour, and the different tags are represented by the differences in the continuity of the blue lines at the ends of the sequence. **a** DNA is extracted and the targeted barcode is amplified from each sample. More than one amplification reaction is performed, each PCR replicate with different tag combinations. Afterwards, double-tagged amplicon products can be pooled and constructed into a sequencing library that is subsequently amplified prior to sequencing. **b** Erroneous sequences are filtered based on (1) identification of unused tag combinations (which may be due to tag jumping), (2) chimeric sequences identification with clustering algorithms, and (3) the sequence copy number and reproducibility across the PCR replicates

A number of DNA metabarcoding toolkits have been developed that include steps to model and detect PCR sequencing errors [20] and chimeric sequences [21]. Additionally, in silico PCR can be used to assess for primer bias [22]. Also, various laboratory methods have been developed to reduce the risk of erroneous sequence assignation in studies based on pooled amplicon datasets [23]. For example, double tagging of amplicons with matching tags is a means for increasing the accuracy of amplicon re-assignment to original samples [8]. A second aspect that has been implemented in laboratory methods is the use of PCR replicates as a means for optimal diversity detection and to discriminate PCR and sequencing artefacts from true biological sequences [24, 25]. This is achieved by filtering out sequences according to copy number and presence across differently tagged PCR replicates (Fig. 1). Amplicons from each PCR replicate are uniquely identifiable in the sequencing output of each sequencing library by the tag combination they carry [26]. The same tag combination can be used for different PCR replicates when pooled into different sequencing libraries, yielding an even bigger scope for the number of samples that can be processed. This highlights the need for the availability of user-friendly tools to sort the sequences according to their tag combinations and across PCR replicates and pools, with same tag combinations possibly occurring in different pools. Furthermore, there is currently no available tool to extract all the relevant information from sequences across PCR replicates for a complete exploration of such datasets in order to take an informed decision on the filtering thresholds for the filtering step. Filtering is one of the most basic and important steps in metabarcoding analyses. A tool developed to address all these issues would be a distinct addition to the currently available tools, which are not specifically designed to deal with this laboratory set up.

**Fig. 2:**
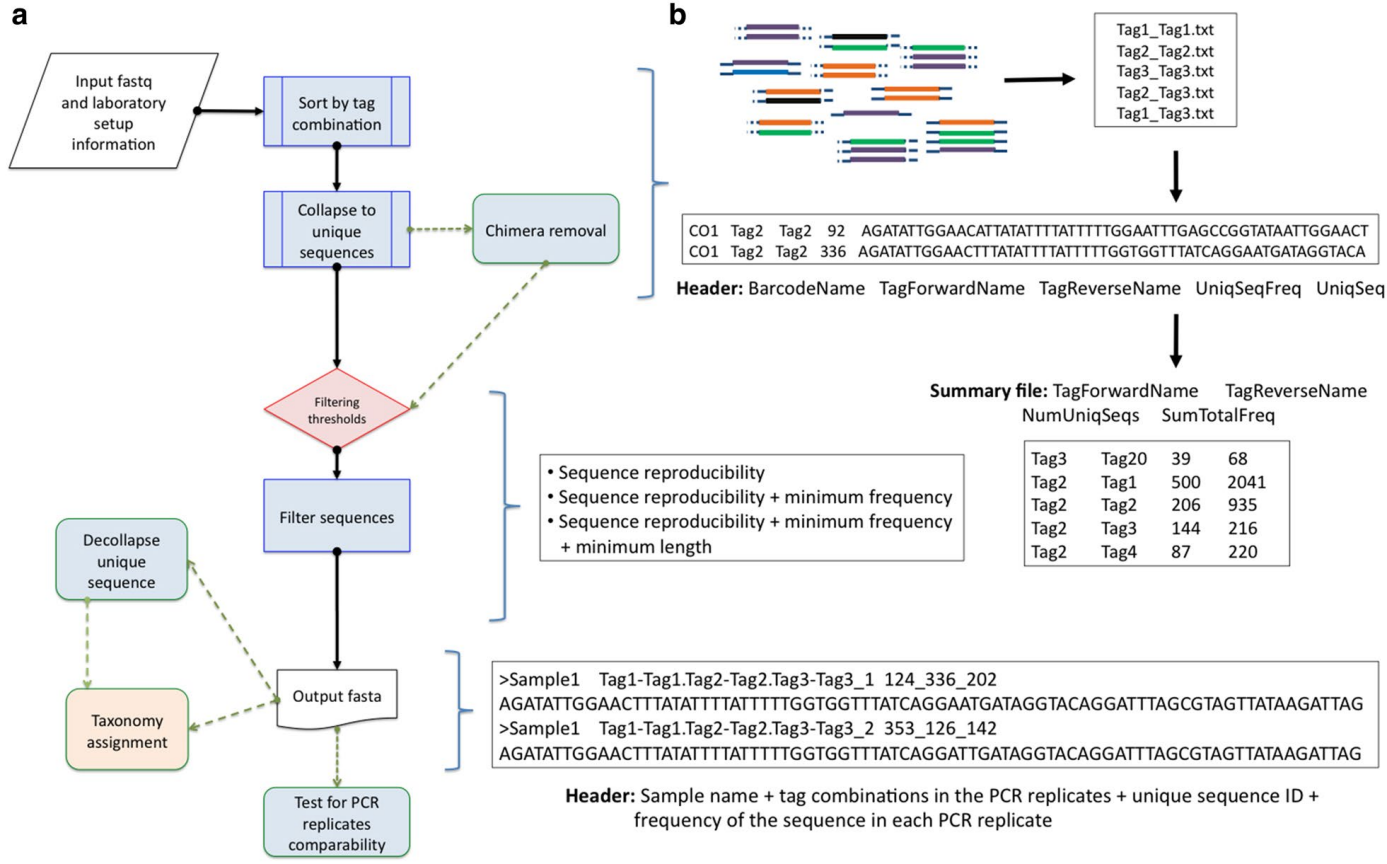
DAMe workflow. **a** DAMe initially sorts sequences by tag combination and collapses the repetitive sequences into unique sequences. An optional chimera removal step can be performed. With the resulting information a decision can be taken on the thresholds for filtering erroneous sequences prior to taxonomy assignment. Renkonen similarity index can be optionally computed for every pair-wise PCR replicate in order to test for the comparability of the PCR replicates. Also, sequences can optionally be de-collapsed if later taxonomy assignment requires this. **b** From each step different output files are obtained, containing information from each sequence such as the sample source, identified tag combination, frequency, and PCR reproducibility

To address the above-mentioned needs, we present DAMe, a standardized metabarcoding toolkit for the straightforward processing of datasets consisting of double-tagged amplicon sequences derived from an unlimited number of samples subject to many PCR replicates and sequencing pools. Specifically, DAMe provides the means to (i) de-multiplex tagged pooled amplicons, (ii) identify sequences with unused tag combinations, which can arise due to e.g. tag jumping events and cross-contamination by tagged primers or tagged amplicons, and (iii) filter out erroneous sequences, which can be due to e.g. contamination, chimera formation, and PCR and sequencing errors. This allows the retrieval of a final output containing unique sequences sorted by used tag combinations from PCR replicates potentially in different pools retaining the information of the sample they derive from and their copy number in each replicate. These sequences are filtered using thresholds regarding the unique sequence’s minimum copy number and reproducibility across the PCR replicates [27]. DAMe also allows the evaluation of the influence of the filtering thresholds on the PCR and sequencing error detection that may impact the taxonomic characterization [28]. DAMe also provides a means to straightforwardly evaluate the total sequence similarity of the PCR replicates, thus their comparability. Overall, due to the simple, yet informative, nature of its output (Fig. 2), DAMe reduces the noise, size, and complexity of metabarcoding sequence datasets, so that they can be easily used for subsequent analyses and taxonomic assignment.

### Datasets

We showcase DAMe using a total of three datasets, hereby referred to as datasets 1, 2, and 3. The samples were collected under licence from Natural England (20122272) and the Home Office (PPL 3002513 and PIL 30/3261). Dataset 1 is a previously published double-tagged dataset generated from 61 Natterers bat (*Myotis nattereri*) faecal pellets [25]. In summary, the dataset consists of ca. 157 bp (excl. primers and tags) arthropod mitochondrial CO1 mini-barcode amplicons. Primers were tagged at the 5′ end [4]. Each DNA extract was independently PCR amplified twice, with each replicate PCR being uniquely labelled by utilizing a different combination of the 5′ tagged forward and reverse primers, e.g. F1-R3, where F1 means forward primer carrying the tag sequence with id 1, and R3 means reverse primer carrying the tag sequence with id 3 (see Additional file 1 for further details).

Dataset 2 (unpublished) consists of COI mini-barcodes amplicons generated from (i) greater horseshoe bat (*Rhinolophus ferrumequinum*) droppings and (ii) bulk insect samples. The primers were 5′ nucleotide tagged [4]. Each DNA extract was independently PCR amplified four times, with each replicate PCR carrying forward and reverse primers with matching tags, i.e. carrying the same tag sequence at both ends, e.g. F1-R1, F2-R2, etc. (see Additional file 1 for further details).

Lastly, we produced a mock dataset as a benchmark to evaluate the results of DAMe (dataset 3). Dataset 3 was constructed by extracting the DNA from ten insects from known taxonomy and from which we also Sanger-sequenced their COI mini-barcodes. Amplification of the COI mini-barcodes was performed as in dataset 2, with matching tag combinations. Four PCR replicates were performed on the insects mixed at equimolar DNA concentrations. See Additional file 1 for an extended description of the generation of this dataset and on the differences between the three datasets.

### DAMe tools

The tools in DAMe were developed to enable to following data processing steps: (i) within pools, double-tagged amplicon sequences are sorted by their tag combination and are collapsed to unique sequences while retaining copy number information, and (ii) across pools and within each sample’s PCR replicates, sequences are filtered by user-defined filtering thresholds (copy number and number of PCR replicates a sequence should occur in). This allows discarding erroneous sequences that have not been produced in a minimum number of the PCR replicates from each sample at a minimum copy number, such as those produced by sequencing and PCR errors and contamination.

DAMe has three extra optional tools can be used for (i) chimera removal, (ii) evaluation of the sequence content similarity in the PCR replicates so that dissimilar PCR replicates such as those that can arise due to laboratory processing errors can be straightforwardly identified, and (iii) decollapsing the collapsed unique sequences. The latter allows for easier integration with other programs that may require the redundancy of the sequences either for taxonomic assignment or other downstream analyses, e.g. [29–32] (Fig. 2a). Additional file 2 contains further details such as command line examples of each tool.

### Input files

DAMe requires as input a fastq file of tagged amplicon sequences that have already been trimmed of adapter and low quality sequences. Reads that were generated using paired-end sequencing must also be merged prior to their input into DAMe, using any of a number of available programs (e.g. [33, 34]). A text file containing information of each tag combination used for each PCR reaction of the samples is also required. All samples must have the same number of replicates. Specifically, this file requires the sample name, the forward tag id, the reverse tag id, and the pool identifier. A second text file must be provided with the name and the sequence of all the tags. And a third text file is also required containing the name of the targeted barcode, followed by the forward and the reverse sequence of the primers. If multiplexed PCRs were performed, the text should include the name and sequences of all the primer pairs used. Examples of the input files are provided in the repository from where DAMe is available. See the Availability of data and materials section and Additional file 2 for further explanations of input files and the structure of the working directory.

### Sorting sequences

With the tool *sort.py,* DAMe initially separates the sequences within each pool based on their tag combination and trims the tag and primer sequences off the amplicon sequence. Post trimming, DAMe collapses the sequences into unique sequences, while retaining information of their prior copy number. Sequences containing errors in the tag/primer sequence are discarded and the number of erroneous sequences is reported (Fig. 2b). Within each pool, text files are created for each tag combination with information on the primers used and the unique sequences along with their copy number. Furthermore, a summary file is generated for each pool, which gives an overview of all tag combinations in the pool along with their unique and total number of sequences. See Additional file 2 for a detailed description of its output files.

The deeper insights obtained solely from the sorting tool include: (i) the distribution of the copy number of the unique sequences, (ii) the abundance characterization of the sequences obtained from negative controls (if included on the laboratory set up), and (iii) identification of sequences with unused tag combinations. To examine these aspects, we first applied *sort.py* on datasets 1 and 2 (Table 1). Although some programs exist for sorting tagged sequences, only a limited number allow processing of double-tagged amplicons [9, 35–37]. However they are not straightforward to apply for sorting and filtering of sequences from datasets consisting of PCR replicates of a same sample that can furthermore be distributed on different pools. See Additional file 1 for an extended discussion on the sorting step.

**Table 1:** Summary of sequence abundance in the sorting and chimera removal steps of DAMe

DAMe information	Number of sequences	
	Dataset 1	Dataset 2–pool 1
Initial input	184,396 total sequences	718,848 total sequences
Sequences with errors in tag/primer	45,932 (24.9 % of total input)	119,619 (16.64 % of total input)
Total sorted sequences	138,464 (75.1 % of total input)	599,229 (83.36 % of initial input)
Total sorted unique sequences	29,952	47,489
Unique sequences from used tags	24,215 (80.85 % of total unique sorted sequences)	36,540 (76.9 % of total unique sorted sequences)
Unique sequences per used tag combination^a^	11; 160.5; 198.5; 1087	1; 552.5; 609; 1896
Frequencies of unique sequences per used tag combination^a^	1; 1; 4.8; 3245	1; 1; 15.47; 32,400
Unique sequences from unused tag combinations	5737 (19.15 % of total unique sorted sequences)	10,949 (23.1 % of total unique sorted sequences)
Unique sequences per unused tag combination^a^	6; 59.5; 98.9; 592	1; 3; 4.7; 640
Frequencies of unique sequences per unused tag combination^a^	1; 1; 3.67; 1740	1; 1; 3.09; 11,699
Chimeric sequences	634 (2.62 % of total unique sequences of used combinations)	1308 (3.6 % of total unique sequences from used combinations)
Unique chimeric sequences per used tag combination^a^	0; 3; 5.2; 61	0; 10; 21.8; 149
Frequencies of unique chimeric sequences per used tag combination^a^	1; 1; 1.36; 21	1; 1; 4.76; 661

^a^ Minimum; median; mean; maximum

### Identification of unused tag combinations

Identification of mistagging patterns can be used to provide more information for accurate filtering of taxonomic diversity [11]. After sorting dataset 1 and 2, for both used and unused tag combinations we examined the number of unique sequences and their frequency through the output files from *sort.py*. Additionally, we analysed the possible tag jumping events in pool 1 from dataset 2. For this we used R v3.1.1 [38] to create a heat map using the copy number of the identified sequences with used as well as unused tag combinations (Fig. 4).

**Fig. 3:**
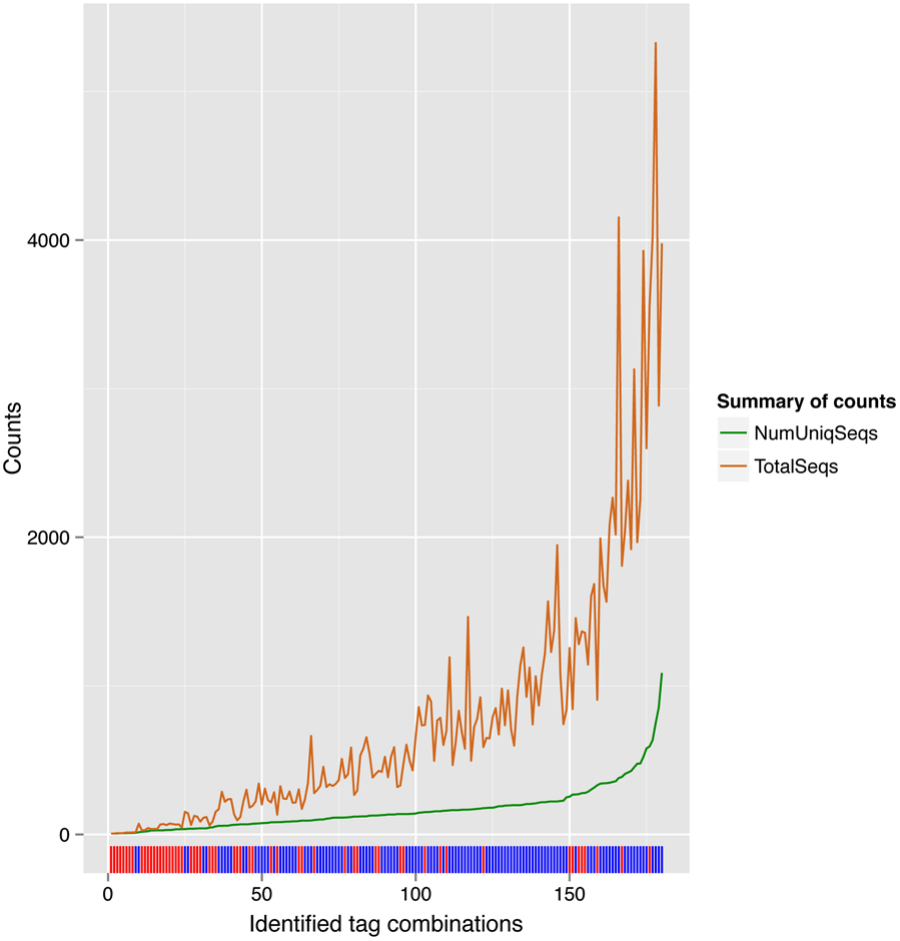
Summary of the sequence content from identified used and unused tag combinations. X-axis are the identified tag combinations. Red ticks on the rug at the X-axis represent unused tag combinations; blue ticks are used tag combinations. The green line is the number of unique sequences (total number of collapsed sequences) and the orange line is the sum of their copy numbers (total number of uncollapsed sequences) from identified used and unused tag combinations in dataset 1

**Fig. 4:**
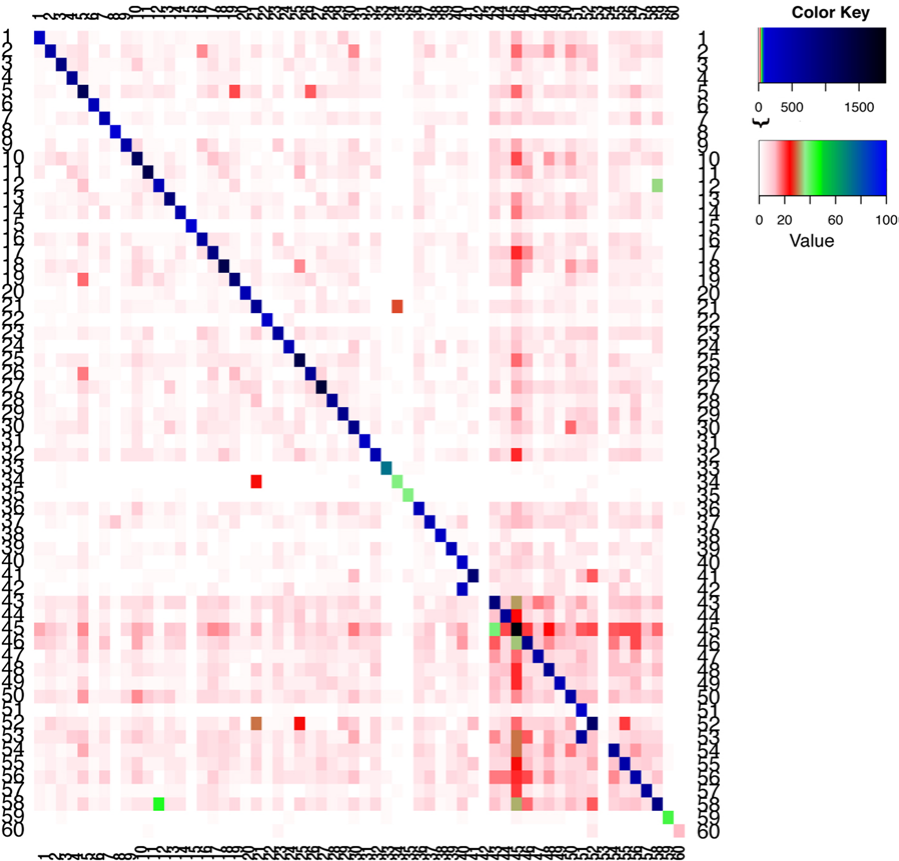
Heat map of the occurrence of tag combinations from dataset 2–pool 1. *Rows* are the forward tags and *columns* are the reverse tags ids. Colours ranging from *white* to *light blue* represent sequence copy numbers from 0 to 100, derived from identified sequences with unused tag combinations. Colours ranging from dark blue to black represent high copy numbers (>100, up to thousands), usually from sequences with the matching tag combinations intended in the laboratory set up

Prior to collapsing the amplicon sequences, the average copy number of each unique sequence from the unused tag combinations was 3 in both datasets; while the average copy number of unique sequences from used tag combinations was 5 and 15 on dataset 1 and 2, respectively. Overall, the unused tag combinations are at low frequencies on dataset 1, although some also contain high frequency sequences (Fig. 3). The 20 forward and 20 reverse tags used in dataset 1 produced sequences with the intended 122 tag combinations, and sequences with 58 unused tag combinations. The 60 forward and 60 reverse tags in pool 1 from dataset 2 produced sequences with the 60 used tag combinations and sequences with 2323 unused tag combinations (Additional file 3). The total number of sequences was clearly higher for sequences with used tag combinations than for sequences with unused tag combinations (Fig. 4).

Another important aspect that can also be easily observed through the use of the sorting tool is the occurrence of some tags instead of others. For example, we observed a strong signal for tag combination F40-R40 and F42-R40, but no sequences carrying the planned tag combination F42-R42. Inspecting the DAMe sorting output across pools, we found this to be a general pattern, indicating that for F42-R42, F42-F40 was actually used. Another similar case appeared for tag combination F51-R51, which seemed to actually be F53-R51. Such mix ups can arise in a number of steps, e.g. during primer synthesis and primer preparation, and are particularly prone to occur when handling large number of samples, PCR replicates, pools and tagged primers. Thus, the sorting tool in DAMe can be used to identify such events to correct the laboratory set up. This is very important, as the backbone of metabarcoding is the reliance on being able to correctly trace tagged sequences back to the PCR replicates and thereby samples that they originated from. See Additional file 1 for deeper discussion on the sequence copy number.

### Chimera identification

We then performed chimeric sequence identification in both datasets in a de novo fashion with *chimeraCheck.py* and characterized the amount of identified chimeras in both datasets. The chimera removal is performed on each pool using UCHIME [21] with default parameters either in a de novo or a reference based approach. This step is highly recommended [39], although not made part of the essential pipeline given that the user might apply other methods for removing chimeras. A total of 634 unique sequences were identified as chimeric in dataset 1 (2.62 % of total unique sequences of used tag combinations), and 1308 in dataset 2 (3.6 % of total unique sequences from used tag combinations) (Table 1). Each of these chimeric sequences had a median frequency of one in both datasets.

### Filtering sequences across PCR replicates for each sample

After sorting the sequences, DAMe can be used to filter out sequences that are assumed to be erroneous with *filter.py*. Although the input fastq for the first step in DAMe has been pre-treated with quality-filtering techniques (such as removal of adapter and low quality sequences), sequencing errors will still remain in the dataset [40–43]. Thus, a filtering step to reduce the presence of erroneous sequences is necessary. This is done under the assumption that erroneous sequences are unlikely to occur multiple times by chance in the separate PCR replicates, and that such sequences are present in low copy numbers, as has been previously shown [12, 44, 45].

This stage requires the user to take an informed decision on the filtering thresholds [30], while ensuring a balanced sequence diversity. This decision is helped from the output files of the sorting step in DAMe. Of particular aid for this decision is the output from sequenced positive and negative controls [24]. Specifically, the filtering thresholds are (i) minimum number of PCR replicates from each sample containing a sequence in order to be retained (i.e. minimum sequence reproducibility, parameter *y*), (ii) the minimum number of copies required for retaining sequences within each PCR reaction, so as to not be considered erroneous (parameter *t*), and (iii) the minimum length of the sequences to be retained (parameter *l*) (Fig. 2b). The filtering is performed for every sample, by comparing the collapsed unique sequences across the PCR replicates, which in turn can be spread across various pools. The thresholds are applied in the next order: (1) reproducibility, (2) minimum copy number, and (3) minimum length.

In order to explore the impact of the filtering thresholds on the amount of filtered sequences, we used the unique sequences from used tag combinations derived after the sorting and chimera removal steps on dataset 1. The thresholds examined include combinations of the following filtering criteria: (i) retention of sequences with a reproducibility of 2/2 and 1/2 (i.e. present in two and one out of the two PCR replicates, respectively), (ii) retention of each unique sequence per PCR with a minimum copy number of one or two, and (iii) retention of only sequences with a minimum length of 157 bp. To this end, we applied *filter.py* with the next parameters: (i) *y* = 1, *t* = 1, (ii) *y* = 1, *t* = 2, (iii) *y* = 2, *t* = 1, and (iv) *y* = 2, *t* = 2.

The filtering parameter with the highest impact on the 23,336 correctly tagged, unique sequences identified post chimera removal in dataset 1, is the minimum number of times a sequence has to be present in each PCR in order to be valid. The second most important parameter is the minimum sequence reproducibility across the PCR replicates (Table 2). See Additional file 1 for an extended discussion on the filtering thresholds.

**Table 2:** Impact of the filtering thresholds on dataset 1

Repr–CopyNum	Sorted clean	Y	y + t	y + t + l^a^
2 PCRs, 2 times	23,336	2472	862	802
2 PCRs, 1 time	23,336	2472	2472	2348
1 PCR, 2 times	23,336	20,864	4195	3995
1 PCR, 1 time	23,336	20,864	20,864	19,647

^a^ y = minimum reproducibility; t = minimum copy number; l = minimum length

### DAMe filtering thresholds benchmarking

Dataset 3 was a mock eDNA sample generated from a laboratory prepared mixture containing known species, at known and equal DNA concentrations, all of which had been CO1 mini-barcoded prior to the experiment. The use of this kind of mock dataset, in which the amplified sequence is known a priori, is useful for detection of error rates and for evaluating filtering strategies [13]. This dataset was therefore an ideal benchmark for calculating the true positive rate (TPR), true negative rate (TNR), false positive rate (FPR), and false negative rate (FNR) of sequences classified as derived from the real sample, or as derived from contamination, or sequencing/PCR errors in the filtering step performed by DAMe with *filter.py*. To this end we clustered with uclust v1.2.22q [46] all the unique sequences from the used tag combinations against our reference database of the 10 insect CO1 sequences. Given the species are from different families or genera, we clustered at 97 % identity. Sequences were classified as derived from the real insect if they clustered to one of the sequences in the database (TP), and classified as derived from contamination or sequencing/PCR errors otherwise (TN). To calculate the different rates, we identified which sequences were kept after the DAMe filtering steps as well as which sequences discarded by DAMe belong to the TP and TN classes. Afterwards, operational taxonomic units (OTUs) were identified on the TP and FP sequences using uclust [46] with 97 % id and OTUs consisting of only one sequence were discarded.

The results show that no filtering at all (reproducibility of 1/4 and minimum copy number of 1) produces the highest TPR, but also the highest FPR (0.37) and the lowest TNR (0.009) (Table 3). Amplicon sequence copy number is known to be inconsistent with specimen counts and biomass, but the use of other variables together with the sequence copy number aids in the analysis of the data [47]. In accordance with this, we observed that the sole inclusion of the minimum copy number filter produces a drastic decline in the FPR (0.076) and increase in the TNR (0.303), however at the cost of lowering the TPR (0.196). While the sole inclusion of the minimum sequence reproducibility has an overall slightly better impact on the results (TPR = 0.211), we found that only a combination of both filtering thresholds causes a more drastic decline on the FPR, with the minimum reproducibility threshold accounting for most of the impact. Another important aspect is that very high filtering thresholds might in fact have a negative impact on the TPR, with the minimum copy number having most of the impact. It is also interesting to note that in spite of the large impact on the number of filtered sequences with even very relaxed filtering thresholds, the actual number of identified insects is kept high, identifying the 10 insects even with strict thresholds such as 4/4 reproducibility and minimum 10 copy number. Thus, in this dataset the importance of the filtering mainly resides on the removed false positives that at later stages of the metabarcoding study can produce taxonomic misidentifications (Table 3).

**Table 3:** Filtering thresholds benchmarking dataset 3

Repr–CopyNum	97 % identity				Correct OTU identifications	Incorrect OTU identifications
	TPR	FNR	FPR	TNR		
1/4–1	0.622	0.000	0.370	0.009	10	111
1/4–2	0.196	0.426	0.076	0.303	10	28
1/4–5	0.063	0.558	0.026	0.352	10	14
1/4–10	0.020	0.601	0.019	0.359	10	12
2/4–1	0.211	0.411	0.069	0.310	10	18
2/4–2	0.092	0.530	0.016	0.363	10	12
2/4–5	0.022	0.599	0.007	0.371	10	6
2/4–10	0.007	0.615	0.005	0.374	10	4
3/4–1	0.112	0.510	0.019	0.359	10	6
3/4–2	0.047	0.574	0.007	0.371	10	4
3/4–5	0.009	0.613	0.004	0.375	10	4
3/4–10	0.005	0.616	0.002	0.377	10	2
4/4–1	0.049	0.572	0.006	0.372	10	3
4/4–2	0.018	0.604	0.004	0.374	10	3
4/4–5	0.005	0.616	0.002	0.376	10	3
4/4–10	0.004	0.618	0.002	0.377	10	2

### PCR replicates similarity

The tool *RSI.py* computes the Renkonen similarity index (RSI) [48] to assess how similar PCR replicates of the same sample are and thus test for the comparability of the PCR replicates of the same sample. This tool allows the user to quickly assess whether any pairs of PCR replicates are completely different indicating that there is an issue in the input file or laboratory set up. Briefly, the RSI is computed based on the frequency of each unique sequence identified in each PCR replicate of each sample. The values range from 0 to 1, where 0 means that the PCR replicates are identical, and 1 means that there is no sequence shared between the PCR replicates. The output file contains the mean RSI of the pair-wise comparison of the replicates per sample. It is also possible to get the RSI of every pair-wise comparison per sample (using the–*explicit* parameter). Although some laboratory setups perform PCR replicates which are then pooled as a means to reduce sequencing costs, these replicates can have the same tag instead of a different tag for each replicate [37]. However, the RSI can only be computed if it is possible to identify the sequences derived from each PCR replicate, meaning that each replicate should have a different tag combination. The examination of the RSI values is an important step given that the PCR replicates should produce comparable sequences and thus justify the use of the sequence reproducibility as a filtering threshold. The RSI can also help to easily pinpoint PCRs that should be considered for repetition.

In order to identify how comparable the PCR replicates are in datasets 1 and 2, and thus confirm the validity of their comparison to support the reproducibility of the sequences, we first used *filter.py* with *y* = 1 and *t* = 1 so that a sequence only had to be present in one PCR replicate and in one copy. Subsequently, we used *RSI.py* to calculate the RSI of every pair-wise PCR replicate comparison of the frequencies of the sequences using an output file from *filter.py*. This allowed us to (i) evaluate the comparability of the PCR replicates, and (ii) obtain a general overview of the reproducibility of the sequences across the PCR replicates.

**Fig. 5:**
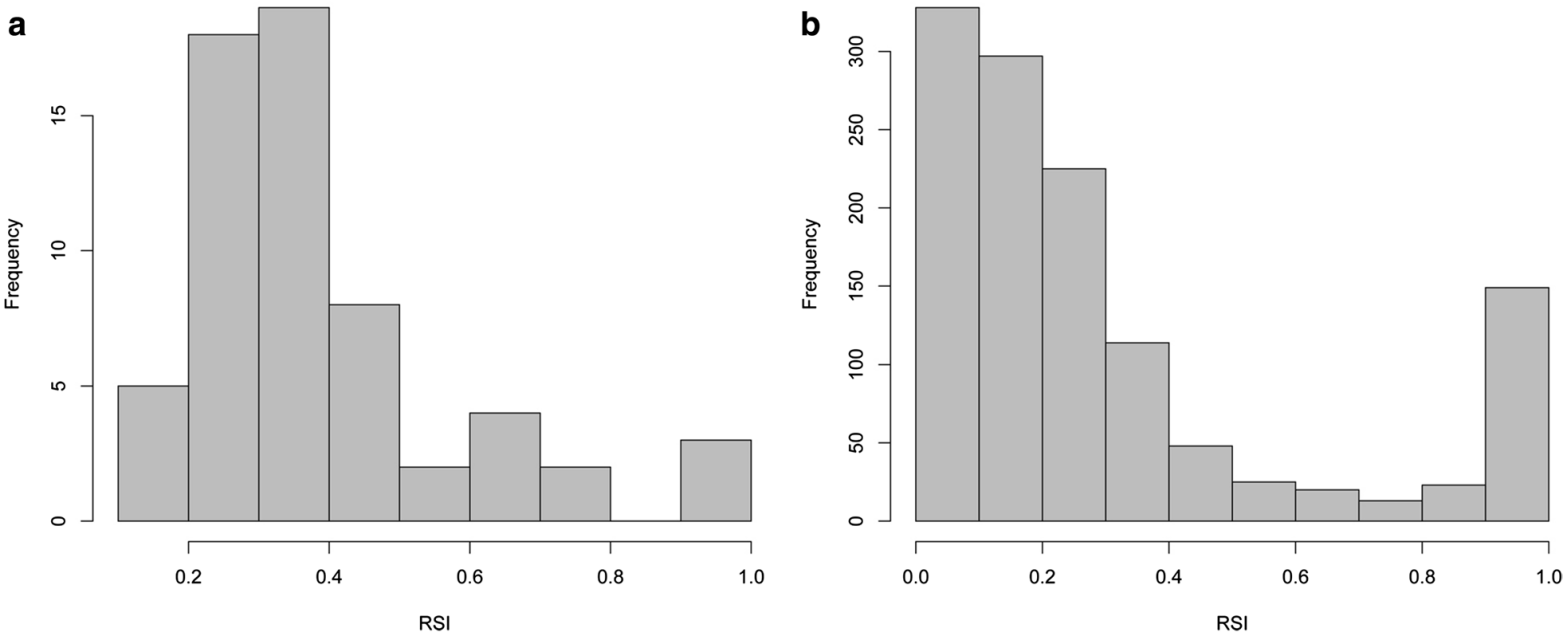
Renkonen similarity index values distributions of the pair-wise comparisons of the PCR replicates from dataset 1 and 2. **a** RSI values distribution of dataset 1. **b** RSI values distribution of dataset 2. A value of 0 means that the sequences are identical, and 1 means that there is no sequence shared between the PCR replicates

Examination of the output from *RSI.py* can be used to easily identify problematic PCR replicates (Additional file 4), for example, an RSI of 0.6 has been previously defined as a threshold for the identification of highly dissimilar replicates [49]. The RSI values are mostly around 0.4, and drop at 0.6 on both datasets (Fig. 5a, b), and in dataset 2 almost all samples with mean RSI equal or very close to 1 are the negative controls (Fig. 5; Additional file 5: Figure S1). The mean RSI and the RSIs from every pair-wise comparison of the four PCRs of the samples in dataset 2 showed that the samples that are not negative controls and that have a large RSI in some of the pair-wise PCR comparisons did not have a large RSI in all the pair-wise comparisons. For example, sample bF1 from dataset 2 had a RSI of 0.34 in the comparisons that did not involve one particular PCR replicate, while all the comparisons involving that particular PCR replicate produced an RSI of 1, showing that there was a mistake in one of the four PCR replicates, which should be repeated or excluded from the analyses. See Additional file 1 for a deeper discussion on the importance of the PCR replicates similarity.

### Sequence reproducibility characterization

Next, we deeper characterized the sequence reproducibility across PCR replicates. To this end, we examined the distribution of the difference of the copy number of each unique sequence prior to applying the filtering thresholds from the used tag combinations between the two PCR replicates in dataset 1. We also looked at the copy number of each unique sequence, taking into account the sequence reproducibility across the PCR replicates. To this end, exemplifying the general pattern observed in the samples, we used a randomly selected sample, pA1, from dataset 2 for deeper examination. Negative controls also provide useful information that can later be used for deciding the filtering thresholds [50], thus we also investigated the sequence content of the extraction blank from the batch of that random sample from dataset 2, Ex_Bl_p1.

**Fig. 6:**
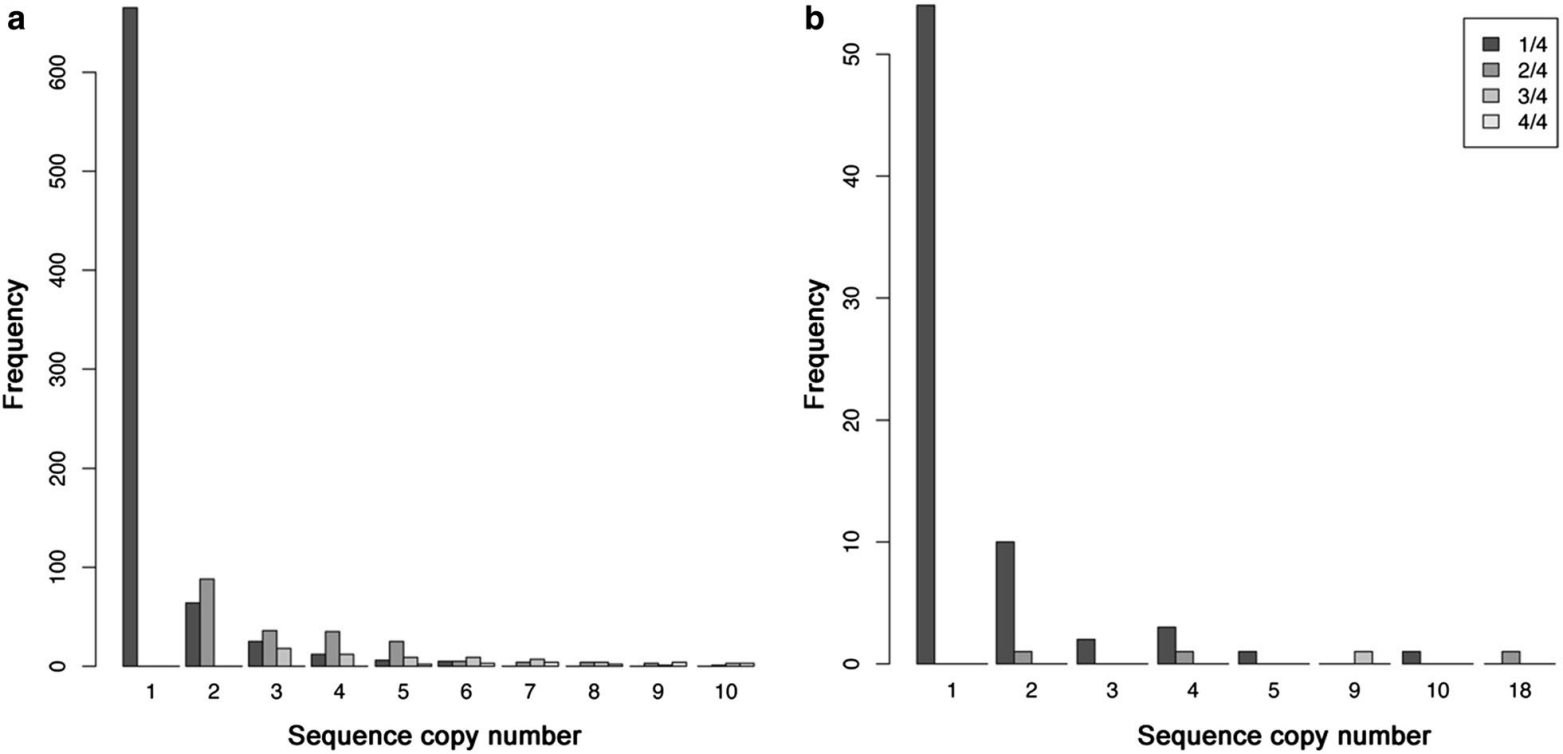
Unique sequence frequency and reproducibility across PCR replicates. **a** Distribution of the abundance of the ten lowest frequencies of the unique sequences in sample pA1 from dataset 2 according to their PCR reproducibility. **b** Distribution of the abundance of the frequencies of the unique sequences in extraction blank Ex_Bl_p1 from dataset 2 according to their PCR reproducibility

We observed that the mean difference of the copy number of each unique sequence across the PCR replicates ranged from 1 to 4 (Additional file 5: Figure S2). The most abundant sequences with a reproducibility of 1/4 are singletons, thus are expected to be erroneous sequences [51]. As copy number increases to 2, the abundance of sequences with reproducibility of 1/4 greatly diminishes. The number of sequences with reproducibility between 1/4 and 1 is more similar at a copy number around 7 (Fig. 6a). In the examined extraction blank, the maximum copy number of a sequence is 18 (16 in one PCR and 2 in another), and besides a single sequence with reproducibility of 3/4 with a total frequency of 9, only sequences with reproducibility index of 1/4 and 2/4 are obtained (Fig. 6b). As observed in the distribution of sample pA1, the abundance of sequences with 1/4 in the extraction blank sample greatly diminishes at a total frequency of two.

The informative output from DAMe assists the challenge of distinguishing low abundance sequences from contaminant or erroneous sequences. The use of positive/negative controls helps to verify the level of sequence detection to guide the selection of the minimum frequency threshold [24] and can be used to detect problems if they are higher than expected [50]. Also the repeated observation of unique sequences across the PCR replicates of a sample helps to distinguish low abundant sequences [52] from erroneous or contaminant sequences [53]. As we observed in dataset 2, the number of unique sequences and their copy number in PCR blanks are very low, and the reproducibility of the sequences is related to their copy number (Fig. 6). See Additional file 1 for an extended discussion regarding the sequence reproducibility parameter.

### Taxonomic identification

Examination of taxonomy assignment on various filtering strategies can be used to evaluate the number of recovered OTUs and the assigned taxonomy [28, 54, 55]. To showcase the use of DAMe together with taxonomy assignment, we analysed in more detail the same previous randomly picked sample pA1 from dataset 2 using the sorted unique sequences without any further filtering. We used blast [56] against the nt database [57] (as in February 2014) and MEGAN v4.70.4 [58] with min. support 1, min score 50, top percent 10, win score 0.0 and min complexity 0.44. In particular, we looked at the number of unique sequences with reproducibility from 1/4 to 4/4, and their percentage of identity to Insecta alone (the target taxon), and to non-Insecta matches only.

**Fig. 7:**
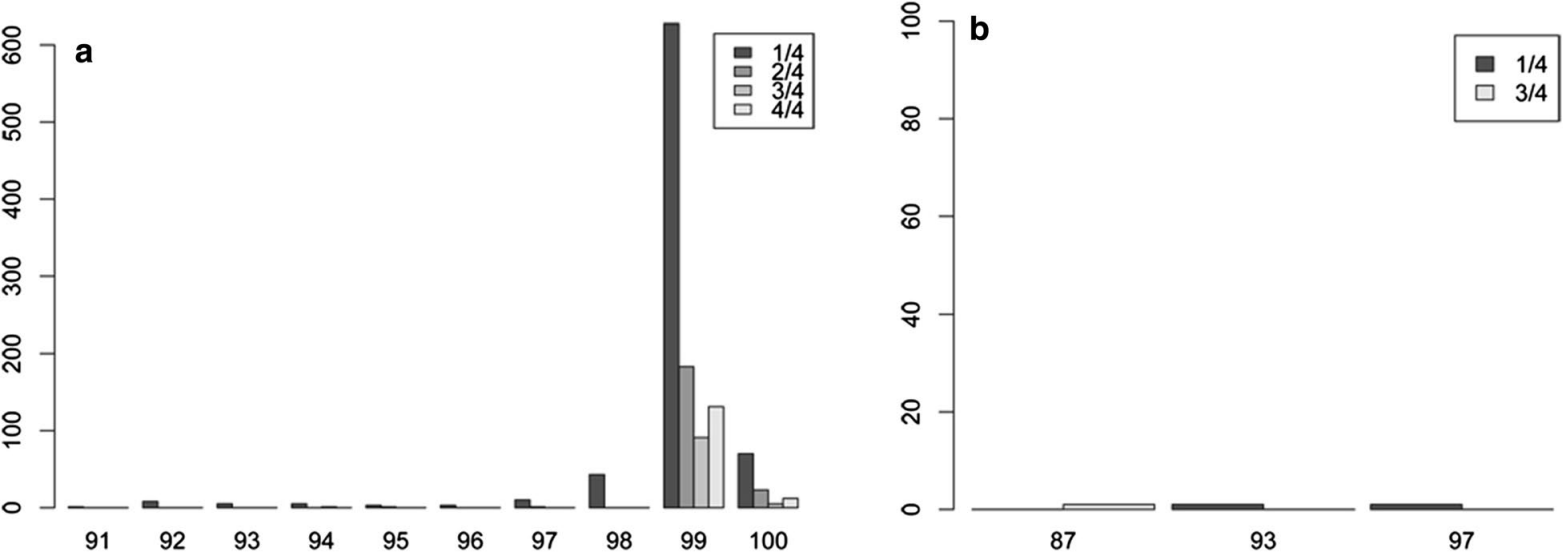
Taxonomic assignment and sequence PCR reproducibility. **a** Percentage of identity of the unique sequences from sample pA1 of dataset 2 with matches to insect reference sequences and their reproducibility. **b** Identity to non-insect matches from the unique sequences from sample pA1 of dataset 2 and their reproducibility

The 1226 unique sequences from sample pA1 from dataset 2 resulted in 34 taxonomic assignations (Additional file 6). We observed that sequences with reproducibility of 1/4 could also have a high similarity to insect reference sequences with the highest abundance at 99 % of sequence similarity. From the hits to species other than insects, we observe that sequences with a reproducibility of 1/4 are the only ones with large (93–97 %) similarity and that sequences with reproducibility of 4/4 have no hits other than insects (Fig. 7). See Additional file 1 for an extended discussion regarding retroactive filtering.

## Conclusions

DAMe is a versatile toolkit to perform the basic, yet critical and informative, sorting and filtering steps of datasets generated with laboratory methods that involve double-tagged amplicons, PCR replicates, and high-throughput sequencing of many pools of samples. DAMe is able to use the information provided by such datasets in order to easily identify sequences carrying unused tag combinations and to guide on the decision of the filtering thresholds. The processing steps included in DAMe are vital for the subsequent taxonomic profiling of the dataset. Given the effective size and complexity reduction of the initial dataset, the final output can be easily handled by the user in order to perform tests without the need of large computational resources.

## Additional files

10.1186/s13104-016-2064-9 Supporting information. This file contains the extended information on the generation of the datasets as well as extended discussions on the main sections of the study.
10.1186/s13104-016-2064-9 DAMe manual. This file contains the manual for DAMe, which describes in detail the parameters, inputs and output files of each tool.
10.1186/s13104-016-2064-9 Tag combinations. This file contains the copy number information of sequences carrying used and unused tag combinations from datasets 1 and 2.
10.1186/s13104-016-2064-9 RSI values. This file contains the RSI values from datasets 1 and 2.
10.1186/s13104-016-2064-9 Supplementary figures. This file contains the supplementary figures.
10.1186/s13104-016-2064-9 Taxonomic identification. This file contains the number of unique sequences from each identified taxon from sample pA1 from dataset 2.

## Abbreviations

HTS: high-throughput sequencing
TP: true positive
FP: false positive
FN: false negative
TN: true negative
TPR: true positive rate
TNR: true negative rate
FPR: false positive rate
FNR: false negative rate
OTU: operational taxonomic unit
