# Supplementary material for: DAMe: a toolkit for the initial processing of datasets with PCR replicates of double-tagged amplicons for DNA metabarcoding analyses
Source: BMC Res Notes. 2016 May 3;9:255. doi: 10.1186/s13104-016-2064-9 (PMC4855357; doi:10.1186/s13104-016-2064-9)
Supplement: Supplementary file 1 — 10.1186/s13104-016-2064-9 Supporting information. This file contains the extended information on the generation of the datasets as well as extended discussions on the main sections of the study. [file 13104_2016_2064_MOESM1_ESM.pdf]

Supporting information

Dataset generation

Dataset 1 was generated with ZBJ-ArtF1c and ZBJ-ArtR2c primers [1] and a set of 20 unique forward and 20 unique reverse primers. Tags were 8 nts long, containing a minimum of three nucleotide mismatches between each other. Post PCR amplification of dataset 1, the double-tagged amplicons were pooled at approximately equimolar ratio. Afterwards, the pool was built into one Illumina sequencing library using the NEBNext DNA Library Prep Master Mix Set for 454 (#E6070) (NEB, Ipswich, MA, USA) although using blunt-end Illumina adapters [2] instead of Roche/454 FLX adaptors. See [3] for further details. The library was subjected to an index PCR post adaptor ligation, followed by standard quality control and purification. The library was sequenced on an Illumina MiSeq platform producing 150 bp paired-end reads. Low quality sequences were trimmed with PRINSEQ v0.20.3 [4] and reads were merged if they overlapped with 100 % identity with an in-house script.

Dataset 2 was generated from i) greater horseshoe bat (*Rhinolophus ferrumequinum*) droppings collected in Gloucestershire, UK (51°2'N, 2°90'W) and ii) bulk insect samples collected in Malaise. As above, the amplicons were generated with insect generic COI mini-barcode primers (ZBJ-ArtF1c and ZBJ-ArtR2c [1]). The primers were 5′ nucleotide tagged [5] to yield a set of 60 unique forward and 60 unique reverse primers. Tags were 7-8 nts long, containing a minimum of three nucleotide mismatches between each other. Each DNA extract was independently PCR amplified four times, with each replicate PCR utilizing forward and reverse primers with matching tags (i.e. carrying the same tag sequence, e.g. F1-R1, F2-R2, etc). Post PCR amplification, the double-tagged amplicons were pooled at approximately equimolar ratio into 14 different pools upon which 14

Illumina sequencing libraries were built as in [3]. The library was subjected to a final index PCR post adaptor ligation, and was sequenced on two MiSeq 250 bp flowcells. The reads were trimmed of adaptor and low quality regions and were merged with AdapterRemoval v1.5.2 [6].

Dataset 3 was generated by extracting the DNA of the next insects as described in [7]: *Pseudotheraptus wayi*, *Dalsira costalis*, *Leptura rubra*, *Ephemeroptera sp.*, *Musca domestica*, *Maculinea arion*, *Trichoptera sp.*, *Chorthippus dorsatus*, *Dysdercus nigrofasciatus*, and *Nezara vididula*. The library was subjected to a final index PCR post adaptor ligation, and was sequenced on MiSeq 250 bp. The reads were trimmed of adaptor and low quality regions and were merged with AdapterRemoval v1.5.2 [6].

Dataset differences and uses

The main differences between the first and the second datasets are that i) every sample in dataset 1 was subject to two replicates while in dataset 2 they were subject to four PCR replicates, and ii) only dataset 2 ensures accurate identification of tag jumping events given that it uses the same tag on both ends [8]. Lastly, different from the two first datasets that derive from real-life studies, from samples with unknown taxa present, dataset 3 contains 10 known taxa from which we know the amplified sequence mini-barcode. This dataset allowed us to benchmark the importance of filtering with DAMe.

Sorting step

A key feature of DAMe is its ability to incorporate an unlimited number of samples into the analysis, allowing the input datasets to be derived from multiple independent sequencing libraries and amplicon pools. This is possible given that DAMe takes into account i) from which sample, ii)

from which PCR reaction, and iii) from which pool each sequence comes from. Even though other programs capable of sorting tagged amplicons can in principle also sort sequences from samples with various PCR replicates divided by pools, the integration of the sorted sequences for further comparison and filtering analyses can only be easy and straightforwardly performed with DAMe, particularly in cases where same tag combinations are repeated on different pools for different samples.

Sequence copy number and reproducibility

Datasets generated with laboratory methods such as the one addressed by DAMe provide a considerable amount of useful information that can be exploited to discriminate between biologically authentic and erroneous sequences. In our identification of sequences with unused tag combinations, we observe that they can be originated from tag jumping events, where such sequences are present at low copy numbers (1 to 30) (Figure 3), and also from unexpected identified laboratory issues (e.g. the identified F42-R42 and F42-R40 tag mix-ups in dataset 2) (Figure 4). By easily identifying such suspicious cases with DAMe, the laboratory set up can be corrected. However, since the average frequency of sequences in many of the intended tag combinations is very low, simple frequency information is not sufficient to discriminate sequences formed by tag jumping or cross contamination of the tag sequences (Suppl Table 1).

Filtering thresholds and retroactive filtering

Overall, the filtering tool in DAMe is exceptional in that it allows an easy and transparent examination of the data and thereby allows the user to make decisions on filtering thresholds for customising processing of different sample types, studies and research questions. A first filtering trial can be run where thresholds should be set so as to retain as many sequences as possible and thereby be able to inspect results for both positive and negative controls and samples. Following this, a new filtering can be performed with informed

and perhaps more conservative thresholds. This will reduce the dataset considerably. Furthermore, the DAMe filtering output files uniquely allow the user to identify if there are any mix-ups either in the lab or in the user-specified DAMe input files (the Tags.txt or PSinfo.txt files). This is achieved through exploration of the filtering output files with regards to reproducibility of sequences between PCR replicates of each sample.

Besides the overview that can be obtained by examining the amount of retained sequences with different thresholds (Table 2), there are various aspects that could aid deciding the thresholds. For example, the decision on the minimum copy number of a sequence can be guided by examining the distribution of the abundance of the copy number of the sequences in negative controls [9], and compare it to the distribution of the frequencies of the sequences from the samples. Those sequences present in the negative controls are presumably derived from contamination. Thus, if cross-contamination is a principal concern, the minimum frequency could be chosen to be the mean or the maximum of the frequencies found in the negative control. The threshold for the sequence reproducibility can also be aided by the information derived from the minimum frequency of the sequences, given that the minimum frequency and the reproducibility are related, with unique sequences present in only one PCR replicate being mostly present only once (Figure 6).

It is also relevant to consider that the examination of assigned taxonomy to the filtered sequences can be used in a retroactive fashion for the filtering of the sequences (Figure 7). Off-target hits may result from erroneous sequences, and examination of the hits to the targeted genera shows the compromise of being strict on the filtering and the obtained taxonomic diversity. The difference in the number of identified taxa depending on the used filtering threshold may be due to retained erroneous sequences from relaxed filtering thresholds yielding taxonomic misidentifications. Alternatively this may indicate the presence of many rare taxa [10]. Regarding taxonomic assignation for comparison of taxa between different samples, it should be noted that (given the use

of matching tag combinations) DAMe enables distinguishing cross-contamination among the samples in a straightforward manner allowing a correct sequence-to-sample assignation.

PCR replicates similarity

The reproducibility of a sequence is directly related to similarity of the PCR replicates of a sample, although primer bias and stochasticity prevent the PCR replicates to contain the exact same diversity and abundance of sequences. The examination of the RSI values is an important step given that the PCR replicates should produce comparable sequences and thus justify the use of the sequence reproducibility as a filtering threshold. The RSI can also help to easily pinpoint PCRs that should be considered for repetition. As an example, the observed raise of sequences with RSI of 1 in dataset 2 correspond to the extraction blanks, in which there is almost no or very few and different sequences to compare (Figure 5).

Sequence reproducibility

One of the most common means for identifying erroneous sequences is the use of *de novo* clustering of the sequences to discard small clusters, which are considered to be erroneous sequences [11,12]. Simple frequency of the sequences is also used to filter sequences [3,9,13]. Avoiding the use of a clustering step to identify molecular operational taxonomic units can be advantageous, given that clustering may be very sensible to the similarity parameters used. Usually, the similarity thresholds are standardized for common markers such as 16S, however, it is also possible to customize barcodes [14] for which there is no benchmarked best similarity threshold.

Tools such as *obiclean* [15] have been developed to aid on the filtering of erroneous sequences based on their frequency and similarity to the most frequent sequences. In spite of these available

tools, there is still a lack of a more systematic and standardized method for detecting the presence of erroneous sequences from datasets of pooled samples and PCR replicates [9]. With the use of DAMe to extract the copy number and reproducibility information from the PCR replicates as an aid in the validation of a sequence, it is possible to take a better informed decision on the filtering thresholds [3,9]. Although abundance information in amplicon datasets cannot be correlated with real abundance in the sample [16], it has been shown that incorrect sequences occur at lower frequencies [17]. We also observed this in our examination of the unique sequence frequency and reproducibility of the sequences in sample pA1 from dataset 2 (Figure 6B).

References

1. Zeale MRK, Butlin RK, Barker GLA, Lees DC, Jones G: **Taxon-specific PCR for DNA barcoding arthropod prey in bat faeces.** *Mol Ecol Resour*. 2011;11(2):236–44.
2. Meyer M, Kircher M: **Illumina sequencing library preparation for highly multiplexed target capture and sequencing.** *Cold Spring Harb Protoc*. 2010;2010(6):pdb.prot5448.
3. Hope PR, Bohmann K, Gilbert MTP, Zepeda-Mendoza M, Razgour O, Jones G: **Second generation sequencing and morphological faecal analysis reveal unexpected foraging behaviour by Myotis nattereri (Chiroptera, Vespertilionidae) in winter.** *Front Zool*. 2014;11(1):39.
4. Schmieder R, Edwards R: **Quality control and preprocessing of metagenomic datasets.** *Bioinformatics*. 2011;27(6):863–4.
5. Binladen J, Gilbert MTP, Bollback JP, Panitz F, Bendixen C, Nielsen R, et al.: **The use of coded PCR primers enables high-throughput sequencing of multiple homolog amplification products by 454 parallel sequencing.** *PLoS One*. 2007;2(2):e197.

6. Lindgreen S: **AdapterRemoval: easy cleaning of next-generation sequencing reads.** *BMC Res Notes*. 2012;5:337.

7. Taylor PJ, Bohmann K, Steyn JN, Schoeman MC, Matamba E, Zepeda-Mendoza ML, et al.: **Bats eat pest green vegetable stinkbugs (Nezara viridula): diet analyses of seven insectivorous species of bats roosting and foraging in macadamia orchards.** *SAMAC Yearb 21*. 2013

8. Schnell I, Bohmann K, Gilbert T: **Tag jumps Illuminated - reducing sequence-to-sample misidentifications in metabarcoding studies.** *Mol Ecol Resour.* 2015. doi: 10.1111/1755-0998.12402

9. De Barba M, Miquel C, Boyer F, Mercier C, Rioux D, Coissac E, et al.: **DNA metabarcoding multiplexing and validation of data accuracy for diet assessment: application to omnivorous diet.** *Mol Ecol Resour*. 2014;14(2):306–23.

10. Huse SM, Welch DM, Morrison HG, Sogin ML: **Ironing out the wrinkles in the rare biosphere through improved OTU clustering.** *Environ Microbiol*. 2010;12(7):1889–98.

11. Mercier C, Boyer F, Bonin A, Coissac E: **SUMATRA and SUMACLUST: fast and exact comparison and clustering of sequences.** *Programs Abstr SeqBio 2013 Work.* 2013;(Abstract):27–9. Available from: http://metabarcoding.org/sumatra

12. Quince C, Lanzen A, Davenport RJ, Turnbaugh PJ: **Removing noise from pyrosequenced amplicons.** *BMC Bioinformatics*. 2011;12:38.

13. Valentini A, Miquel C, Nawaz MA, Bellemain E, Coissac E, Pompanon F, et al.: **New perspectives in diet analysis based on DNA barcoding and parallel pyrosequencing: the trnL approach.** *Mol Ecol Resour*. 2009;9(1):51–60.

14. Taylor PG: **Reproducibility of ancient DNA sequences from extinct Pleistocene fauna.** *Mol Biol Evol*. 1996;13(1):283–5.

15. Boyer F, Mercier C, Bonin A, Taberlet P, Coissac E: **OBITools: a Unix-inspired software package for DNA metabarcoding**. *Mol Ecol Resour*. 2015. doi: 10.1111/1755-0998.12428

16. Deagle BE, Thomas AC, Shaffer AK, Trites AW, Jarman SN: **Quantifying sequence proportions in a DNA-based diet study using Ion Torrent amplicon sequencing: which counts count?** *Mol Ecol Resour*. 2013;13(4):620–33.

17. Reeder J, Knight R: **Rapidly denoising pyrosequencing amplicon reads by exploiting rank-abundance distributions.** *Nat Methods*. 2010;7(9):668–9.
