# Supplementary material for: DAMe: a toolkit for the initial processing of datasets with PCR replicates of double-tagged amplicons for DNA metabarcoding analyses
Source: BMC Res Notes. 2016 May 3;9:255. doi: 10.1186/s13104-016-2064-9 (PMC4855357; doi:10.1186/s13104-016-2064-9)
Supplement: Supplementary file 2 — 10.1186/s13104-016-2064-9 DAMe manual. This file contains the manual for DAMe, which describes in detail the parameters, inputs and output files of each tool. [file 13104_2016_2064_MOESM2_ESM.pdf]

DAMe v1.0

DESCRIPTION

DAMe is a set of python scripts designed to perform the first steps in the analysis of very complicated datasets generated by metabarcoding laboratory methods that use double-tagged amplicons, PCR replicates, multiplexing, pooling samples, and sequencing with HTS technologies.

DAMe makes no assumptions on the used sequencing platform, and it is able to detect tag-jumping events, chimeric sequences, sequencing errors, and contamination. DAMe is a toolkit of python scripts that serve as tools to perform the first cleaning steps of a metabarcoding amplicon dataset following the next pipeline:

1.- Sort sequences from pooled samples by tag combination and collapse to unique sequences
2.- Optionally, remove chimeras using UCHIME in a de novo or reference-based fashion.
3.- Filter sequences that contain PCR/sequencing errors, chimeric sequences, and sequences arising from contamination.
4.- Optionally, the user can decollapse the unique sequences.

The final output can be directly used for any other program for taxonomic profiling.

INSTALLATION

DAMe is a set of python scripts that does not need any sort of compilation. It was developed in Python 2.7.3 and uses the following modules which are generally already installed in the user's system:

- re
- os
- sys
- subprocess
- string
- argparse
- optparse

Make sure to put DAMe's bin in your path. Alternatively, provide the full path when running the programs, making sure that the modules are in the same directory.

INPUT FILES

DAMe requires four types of input files: fastq sequence file(s) and three text files containing information on tags, primers and an overview of tag combinations for each sample's PCR replicate. The fastq-file should contain double-tagged amplicon sequences that have been trimmed of adapter and low quality sequences. Reads that were generated using paired-end sequencing must be merged prior to their input into DAMe, using any of a number of available programs. The tag input file (Tags.txt) should contain the number and the sequence of all the tags and the primer file (Primers.txt) should contain the name of the primer set and/or the targeted marker as well as the forward and the reverse sequence of the primers (5'-3'). A text file containing information of each tag combination used for each PCR reaction of each samples is also required (PSinfo.txt). Specifically, it requires the sample name, the forward tag number, the reverse tag number, and the pool number.

Examples of the required files are shown next:

#########
Tags.txt
#########

TCTGCGAG	Tag1
ATCAGCAG	Tag2
ATACAGTC	Tag3
ATCATATC	Tag4

#########
Primers.txt
#########

CO1	AGATATTGGAACWTTATATTTTATTTTTGG	WACTAATCAATTWCCAAATCCTCC

#########
PSinfo.txt (PCRsetsInfo.txt)
#########

#An example of a PSinfo.txt for the processing of 5 samples, with 2 PCR replicates, pooled into only one sequencing library.

Sample1	Tag2	Tag4	1
Sample1	Tag3	Tag3	1
Sample2	Tag2	Tag12	1
Sample2	Tag3	Tag11	1
Sample3	Tag4	Tag19	1
Sample3	Tag5	Tag17	1
Sample4	Tag2	Tag5	1
Sample4	Tag3	Tag5	1
Sample5	Tag4	Tag5	1
Sample5	Tag5	Tag5	1

WORKING DIRECTORY STRUCTURE

DAMe requires the input files to be placed within a user specified working directory in which files are structured as shown below. The shown example is for three pools, but DAMe can be used for an unlimited number of pools. Next we show an example of a study that uses three pools.

```
| - Working directory
        |  - Primers.txt
        |  - Tags.txt
        |  - PSinfo.txt
        |  - pool1
        |         |- Pool1_merged.fastq
        |  - pool2
        |         |- Pool2_merged.fastq
        |  - pool3
                  |- Pool3_merged.fastq
```

USAGE

#########
sort.py
#########

Usage

sort.py [-h] -fq FQ -p P -t T [--keepPrimersSeq]

Sort amplicon sequences tagged on each end by tag combination

Optional arguments:

- -h, --help Show this help message and exit
- -fq FQ Input fastq with amplicon sequences
- -p P Input text file with primer name and forward and reverse sequences [Format: Name ForwardSeq ReverSeq]
- -t T Input text file with tag ids and sequences [Format: TagSeq TagId]
- --keepPrimersSeq Use this parameter if you want to keep the primer sequences from the amplicon instead of trimming it [default not set]

Example:

python sort.py -fq Paul.AllOneLine.fastq -p Primers.txt -t Tags.txt

An example of the necessary input text files can be found in the "example" directory.

Output

This program produces two types of output. For each identified tag combination a file named with the used tags is created containing the next tab separated fields: barcode name, forward tag id, reverse tag id, frequency of that sequence, sequence.

Example:

```
CO1	Tag7	Tag8	1
	AGATATTGGAACATTATATTTTATTTTTGGGATTTGAGCTGGAATAGTAGAAACCTCTTTAAGA
TTATTAATTCGAGCTGAATTAGGAAATCCTGGATCATTAATTGGAGATGATCAAATTTATAATACTATT
GTCACAGCACATGCTTTTATTATAATTTTTTTTATAGTTATACCTATTATAATTGGAGGATTTGGAAAT
TGATTAGTA
```

Also a file called "SummaryCounts.txt" is created which contains the next tab-separated fields: forward tag id, reverse tag id, total number of unique sequences, sum of the frequencies of the unique sequences.

Example:

```
Tag3	Tag20	39	68
```

If having more than one pool, sort.py is run for each pool. Within each pool, separate text files are created for each tag combination. Each of these reports the primer set/marker used and lists the unique sequences along with their copy number. Furthermore, for each pool a summary file is generated, which gives an overview of all tag combinations in the pool along with their unique and total number of sequences.

###############
chimeraCheck.py
###############

Usage

chimeraCheck.py [-h] -psInfo PSINFO -x X [-p P]

Create necessary files to operate on sequences per PCR reaction

Optional arguments:

- -h, --help	Show this help message and exit

- -psInfo PSINFO Text file with the information on the tag combination in each PCR reaction for every sample [Format: sampleName TagIdForwardFromPCR1 TagIdReverseFromPCR1 Pool# sampleName TagIdForwardFromPCR2 TagIdReverseFromPCR2 Pool# ...]
- -x X Number of PCR replicates performed per sample
- -p P The number of pools in which the samples were divided for sequencing (in case of tag combinations repetition due to processing so many samples) [default 1] NOTE: If using pools, each fastq must be in a folder called pool#, in which the sort.py was run for each pool inside the corresponding folder, and this program chimeraCheck.py is run in the parent directory of the pools directories

Example:

python chimeraCheck.py -psInfo PCRsets_info.txt -x 2

An example of the necessary text files can be found in the "example" directory. "PCRsets_info.txt" is an example of a laboratory set up that only used one pool for 61 different samples (dataset 1 from the paper). "PCRsetsInfo_pools.txt" is an example for when there is more than one pool. This file is the first part of the one used for dataset 2 in the paper.

Output

This program produces two types of output. One type is similar to one of the outputs from the sort.py tool, in which the tag ids and the frequency is reported for each unique sequence after removing chimeras. The file name is the id of the tags, and the suffix noChin.txt (e.g. Tag5_Tag15.noChim.txt)

Example:

CO1 Tag5 Tag15 111
AGATATTGGAACATTATATTTTATTTTTGGGGCGTGGTCGGGCGCGGTAGGTATAGCTTTAAGCATAATTATCCGAACAGAGCTAGGTCATGCCGGGAGATTAATTGGAGACGATCAAATTTATAATGTAATTGTTACTGCACATGCTTTTGTAATAATTTTCTTTATAGTGATACCTATTATAATTGGAGGATTTGGAAATTGATTAGTT

The second type of output is 4 different fasta files. The next fasta files are produced: One fasta file for each pool in which all the unique sequences are contained, and that is going to be sorted to produce a second fasta file in order to be used for removing chimeras. The step of removing chimeras produces two files, one containing the identified chimeric sequences and the ther containing only the non-chimeric sequences.

The headers of the fasta files contan the next information: barcode name, forward tag id, reverse tag id, the number of times that sequence is found (its frequency).

Example:

```
>CO1_Tag2_Tag4_1;size=3
AGATATTGGAACATTATATTTTATTTTTGGAGTTTGATCTGCTATAGCAGGAACTGCTATAAGAGTATT
AATTCGAATAGAGTTAGGAAATCCTGGAAGATTATTAGGAGATGATCATTTATATAATGTAGTGGTTAC
TGCTCATGCTTTTGTTATAATTTTTTTTATAGTTATACCTATTATAATTGGAGGATTTGGAAATTGATT
AGTT
```

##########
filter.py
##########

Usage

filter.py [-h] -psInfo PSINFO [-x X] [-y Y] [-p P] [-t T] [-l L] [--chimeraChecked]

Filter the multiplexed sequences by the presence of the sequence in the different PCR reactions at a given minimum abundance and a minimum length

Optional arguments:

- -h, --help Show this help message and exit
- -psInfo PSINFO Text file with the information on the tag combination in each PCR reaction for every sample [Format: sampleName TagIdForwardFromPCR1 TagIdReverseFromPCR1 Pool# sampleName TagIdForwardFromPCR2 TagIdReverseFromPCR2 Pool# ...]
- -x X Number of PCR replicates performed per sample
- -y Y Number of PCR replicates in which the sequence has to be present
- -p P The number of pools in which the samples were divided for sequencing (in case of tag combinations repetition due to processing so many samples) [default 1] NOTE: If using pools, each fastq must be in a folder called pool#, in which the sort.py was run for each pool inside the corresponding folder, and this program is run in the parent directory of the pools directories
- -t T Minimum number of times a unique sequence has to be present
- -l L Minimum sequence length
- --chimeraChecked Use this parameter if you have performed a chimera check on the sorted collapsed sequence files [default not set]

Example:

python filter.py -psInfo PCRsets_info.txt -x 2 -y 2 -t 2 -l 211 -p 1 --chimeraChecked

An example of the necessary text files can be found in the “example” directory. “PCRsets_info.txt” is an example of a laboratory set up that only used one pool for 61 different samples (dataset 1

from the paper). "PCRsetsInfo_pools.txt" is an example for when there is more than one pool. This file is the first part of the one used for dataset 2 in the paper.

Output

This tool produces two types of output files: comparison files and fasta files. The fasta files are the fastas of every comparison step for which there is a tab-separated comparison file. The next comparison files are created:

1.- A text file with the information of every unique sequence across the different PCR replicates is first created, called e.g "Comparisons_2PCRs.txt". The tab-separated fields contain the next information: sample name, ids of the tags used in each PCR replicate, the frequency of the sequence in each PCR replicate, the sequence.

Example:

Sample1	Tag2-Tag4	0	Tag3-Tag3	1
	AGATATTGGAACATTATATTTTATTTTTGGAGTTTGGTCTGCTATAGCAGGAACAGCTATAAGA
GTATTAATTCGAATAGAGTTAGGAAATCCTGGAAGATTATTAGGAGATGATCATTTATATAATGTAGTG
GTTACTGCTCATGCTTTTGTTATAATTTTTTTATAGTTATGCCTATTATAATTGGAGGATTTGGAAAT
TGATTAGTA

2.- A second comparison file is created in which the records not passing the minimum reproducibility threshold are removed (e.g. "Comparisons_2outOf2PCRs.txt")

3.- Then the minimum frequency threshold is applied (e.g. "Comparisons_2outOf2PCRs.countsThreshold2.txt").

Finally, the minimum length threshold is applied and a fasta file called "FilteredReads.fna" is created. This is the last created file that should be used for any subsequent analysis. The header contains the next tab-separated information: sample name, ids of the tags used on each PCR replicate, a unique sequence id, the frequencies of the sequence in each PCR replicate.

Example:

>Sample1	Tag2-Tag4.Tag3-Tag3_7	3_11
AGATATTGGAACATTATATTTTATTTTTGGAGTTTGATCTGCTATAGCAGGAACTGCTATAAGAGTATT
AATTCGAATAGAGTTAGGAAATCCTGGAAGATTATTAGGAGATGATCATTTATATAATGTAGTGGTTAC
TGCTCATGCTTTTGTTATAATTTTTTTATAGTTATACCTATTATAATTGGAGGATTTGGAAATTGATT
AGTT

A similar fasta file is created for each of the comparison files previously described.

#############
decollapse.py
#############

Usage

python decollapse.py [-h] -input INPUT [-outFas OUTFAS]

This program repeats X number of times a sequence reported like this: Primer Tag1 Tag2 Freq Seq Qual Header. The output is a fasta withe >Tag1.Tag2.Freq_RN , where RN is the writen line number in the output.

Arguments:

- -h, --help Show this help message and exit
- -input INPUT Text file with the information on the tag combination and freq of each unique seq
- -outFas OUTFAS Output fasta with the unique sequences being repeated as many times as their reported freq [default "Decollapsed.fasta"]

Example:

python decollapse.py -input Tag5_Tag8.txt -outFas Tag5_Tag8.decollapsed.fasta

Output

This tool outputs only one fasta file containing each unique sequence repeated as many times as its frequency. The header information is: ids of the tags used on each PCR replicate, total frequency f that sequence, a unique sequence entry identifier.

Example:

```
>Tag7-Tag15.Tag9-Tag15.2_1
AGATATTGGAACTTTATATTTTATTTTTGGTGGTTTATCAGGAATGATAGGTACAGGATTTAGCGTAGT
TATAAGATTAGAATTATCTAACCCAGGAGATTTATATTTAGCAGGAGATTATCAATTATATAATGTAAT
TATAACTGCTCACGCTTTTATTATGATTTTCTTCTTAGTTATGCCTGTATTAATAGGAGGATTTGGTAAT
TGATTAGTA
>Tag7-Tag15.Tag9-Tag15.2_2
AGATATTGGAACTTTATATTTTATTTTTGGTGGTTTATCAGGAATGATAGGTACAGGATTTAGCGTAGT
TATAAGATTAGAATTATCTAACCCAGGAGATTTATATTTAGCAGGAGATTATCAATTATATAATGTAAT
TATAACTGCTCACGCTTTTATTATGATTTTCTTCTTAGTTATGCCTGTATTAATAGGAGGATTTGGTAAT
TGATTAGTA
>Tag7-Tag15.Tag9-Tag15.3_3
AGATATTGGAACTTTATATTTTATTTTTGGTGGTTTATCAGGAATGATAGGTACAGGATTTAGCGTAGT
TATAAGATTAGAATTATCTAACCCAGGAGATTTATATTTAGCAGGAGATTATCAATTATATAATGTAAT
TATAACTGCTCACGCTTTTATTATGATTTTCTTCTTAGTTATGCCTGTATTAATAGGAGGATTTGGAAAT
TGATTAGTA
>Tag7-Tag15.Tag9-Tag15.3_4
AGATATTGGAACTTTATATTTTATTTTTGGTGGTTTATCAGGAATGATAGGTACAGGATTTAGCGTAGT
TATAAGATTAGAATTATCTAACCCAGGAGATTTATATTTAGCAGGAGATTATCAATTATATAATGTAAT
```

TATAACTGCTCACGCTTTTATTATGATTTTCTTCTTAGTTATGCCTGTATTAATAGGAGGATTTGGAAAT
TGATTAGTA
>Tag7-Tag15.Tag9-Tag15.3_5
AGATATTGGAACTTTATATTTTATTTTTGGTGGTTTATCAGGAATGATAGGTACAGGATTTAGCGTAGT
TATAAGATTAGAATTATCTAACCCAGGAGATTTATATTTAGCAGGAGATTATCAATTATATAATGTAAT
TATAACTGCTCACGCTTTTATTATGATTTTCTTCTTAGTTATGCCTGTATTAATAGGAGGATTTGGAAAT
TGATTAGTA

#############
RSI.py
#############

Usage

python RSI.py [-h help] [-e explicit] [-o output FILE] <in.txt>

Computes the Renkonen Similarity Index or Percentage Similarity Index between pairs of PCR replicates. The output file contains the means for the RSI values belonging to the same sample.

Optional arguments:

- -h, --help Show this help message and exit
- -e, --explicit For experiments with more than two replicates, the output can contain the RSI value for every pairwise comparison [default not set]
- -o FILE, --output=FILE Output file name. If no file is provided, the output will be directed to 'RSI_output.txt'.

Example:

python RSI.py --explicit Comparisons_4PCRs.txt

An example of the necessary text file can be found in the "example" directory with the name Comparisons_4PCRs.txt.

Output

This program can produce two different kinds of output files. The default output is a file with two tab-separated columns. The first column corresponds to the sample names and the second one to the mean of the RSI values of every pairwise comparison made between replicates from that specific sample.

Example:

Sample	RSI
X4	0.966666666667

pD9	0.347822352442
pD8	0.286781492997
pD7	0.665882105185
pD6	0.394603124201
pD5	0.611411834584

The alternative format of the output file is the ‘explicit’, option –e or --explicit. The difference with the previous one is that the means are not calculated. The RSI value of every pairwise comparison between two PCR replicates is explicitly shown in the output file.

Example:

Sample	ReplicateA	ReplicateB	RSI
X4	1	2	1.0
X4	1	3	1.0
X4	1	4	1.0
X4	2	3	1.0
X4	2	4	1.0
X4	3	4	0.8
pD9	1	2	0.299253880574
pD9	1	3	0.355540766835
pD9	1	4	0.352368315049
pD9	2	3	0.300623906013
pD9	2	4	0.379322533242
pD9	3	4	0.399824712943

CITE

Zepeda Mendoza, *et al.,* (2015) *"DAMe: A Toolkit for the Initial Filtering Steps on complex Datasets from Double-tagged Amplicons for DNA Metabarcoding Analyses"*

CONTACT

For any enquiries, correspondence is sent to lisandracady@gmail.com
