# Supplementary material for: DAMe: a toolkit for the initial processing of datasets with PCR replicates of double-tagged amplicons for DNA metabarcoding analyses
Source: BMC Res Notes. 2016 May 3;9:255. doi: 10.1186/s13104-016-2064-9 (PMC4855357; doi:10.1186/s13104-016-2064-9)
Supplement: Supplementary file 5 — 10.1186/s13104-016-2064-9 Supplementary figures. This file contains the supplementary figures. [file 13104_2016_2064_MOESM5_ESM.pdf]

Supplementary figures

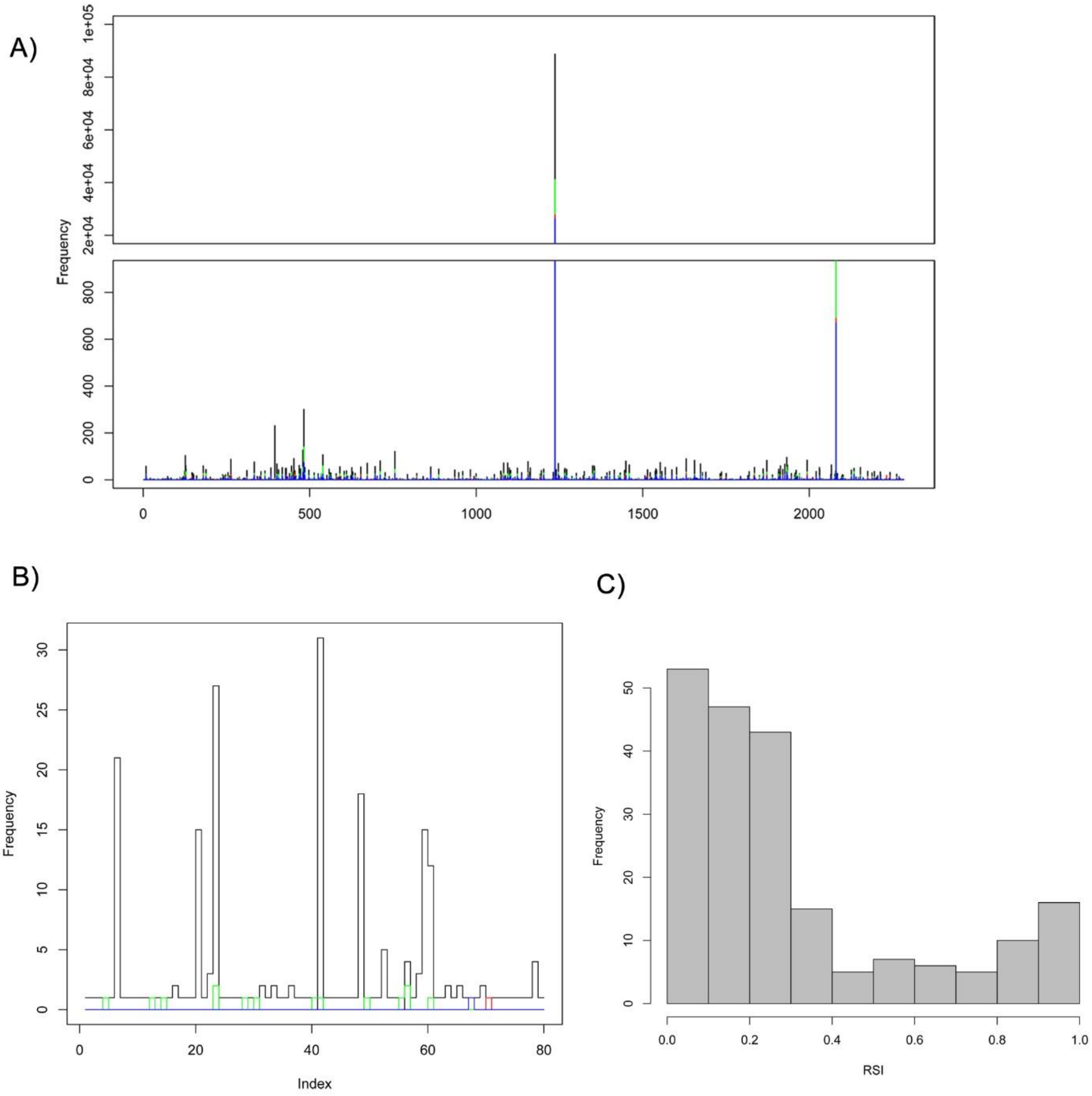

Figure S1 Renkonen similarity index of the pair-wise comparisons of the PCR replicates. A) Sequence frequency comparison of the sample with the lowest RSI in dataset 2 (zC1 with RSI=0.03). X-axis are unique sequences. B) Sequence frequency comparison of the sample with the largest RSI in dataset 2 (X3 with RSI=1). X-axis are unique sequences. C) Mean RSI distribution of the pair-wise comparisons of the 4 PCR replicates of samples from dataset 2.

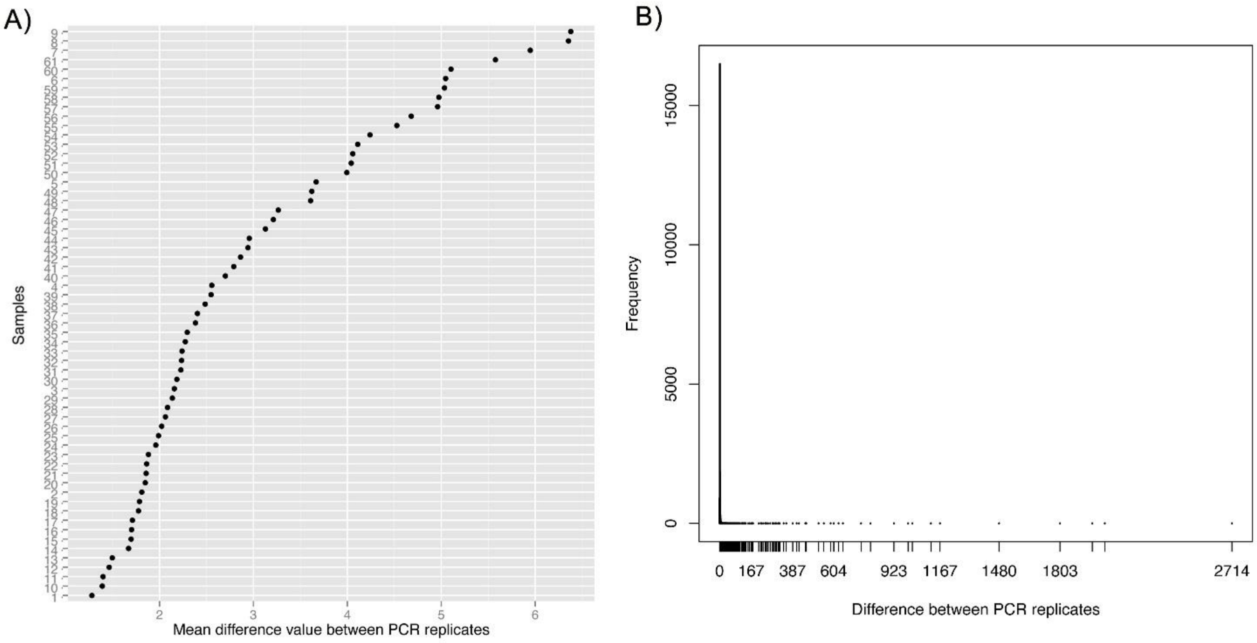

Figure S2 Sequences copy numbers in PCR replicates. A) Mean difference of the copy number counts of each unique sequences across the PCR replicates in each sample in dataset 1. To obtain this we calculated for each unique sequence the copy number difference of each PCR replicate in a pair-waise comparison and obtained the mean value. B) Frequency of the differences of the copy number counts on the two PCR replicates of dataset 1.
